# Characterization of the complete chloroplast genome of *Cynanchum acutum* subsp. s*ibiricum* (Apocynaceae)

**DOI:** 10.1080/23802359.2023.2256496

**Published:** 2023-09-19

**Authors:** Erdong Zhang, Yi Liu, Yan Wang, Xuedan Zhang, Yuqing Wei, Lei Zhang

**Affiliations:** a Key Laboratory of Ecological Protection of Agro-pastoral Ecotones in the Yellow River Basin National Ethnic Affairs Commission of the People’s Republic of China, School of Biological Science & Engineering, North Minzu University, Yinchuan, Ningxia, P. R. China; b Operation Management Department, Yinchuan Hedong International Airport, Yinchuan, Ningxia, P. R. China

**Keywords:** Chloroplast genome, apocynaceae, *cynanchum acutum* subsp. *sibiricum*, phylogenetic analyses

## Abstract

In this study, we assembled the complete chloroplast (cp) genome of *Cynanchum acutum* subsp. *sibiricum* using high-throughput Illumina sequencing reads. The resulting chloroplast genome assembly displayed a typical quadripartite structure with a total length of 158,283 bp, which contained a pair of inverted repeat regions (IRs) of 24,459 bp. These two IRs were separated by a large single-copy region (LSC) and a small single-copy region (SSC) of 89,424 bp and 19,941 bp in length, respectively. The *C. acutum* subsp. *sibiricum* cp genome contained 130 genes, and its overall GC content was 37.87%. Phylogenetic analysis among *C. acutum* subsp. *sibiricum* and nine other *Cynanchum* species demonstrated that *C. acutum* subsp. *sibiricum* was closely related to *C. chinense*. The *C. acutum* subsp. *sibiricum* cp genome presented in this study lays a good foundation for further genetic and genomic studies of the *Cynanchum* as well as Apocynaceae.

## 1. Introduction

*Cynanchum acutum* L. subsp. *sibiricum* (Willd.) K. H. Rech 1970 (Rechinger, 1970), a medicinal plant, belongs to Apocynaceae (Li et al. 1995). It is distributed in the northwest of China, including Inner Mongolia, Ningxia, Gansu and Xinjiang (Li et al. 1995). The dried whole plant of *C. acutum* subsp. *sibiricum* was used as a traditional Chinese medicine to treat bleeding and inflammation (Liu et al. 2007). This medicinal plant has been used for the therapy of chronic tracheitis in China (Yildiz et al. 2017).

Recent usage of the complete chloroplast genome in solving phylogenetic problem has been on the rise due to its unique nature and its capability to unravel valuable information worthy of adopting (Abba et al. 2021). Complete chloroplast genomes have been used vividly in resolving some unanswered questions in plant taxonomy recently (Ruhsam et al. 2015). In addition to the evolutionary perspective, the chloroplast genome has important implications in chloroplast transformation (Daniell et al. 2008). A systematic study of *C. acutum* subsp. *sibiricum* would have significant implications for understanding the origin and evolution of the *Cynanchum* and local flora. However, the chloroplast genome of *C. acutum* subsp. *sibiricum* has not been reported.

In this study, the complete chloroplast genome of *C. acutum* subsp. *sibiricum* has been assembled in order to lay a foundation for further research.

## 2. Materials

**Figure 1.**
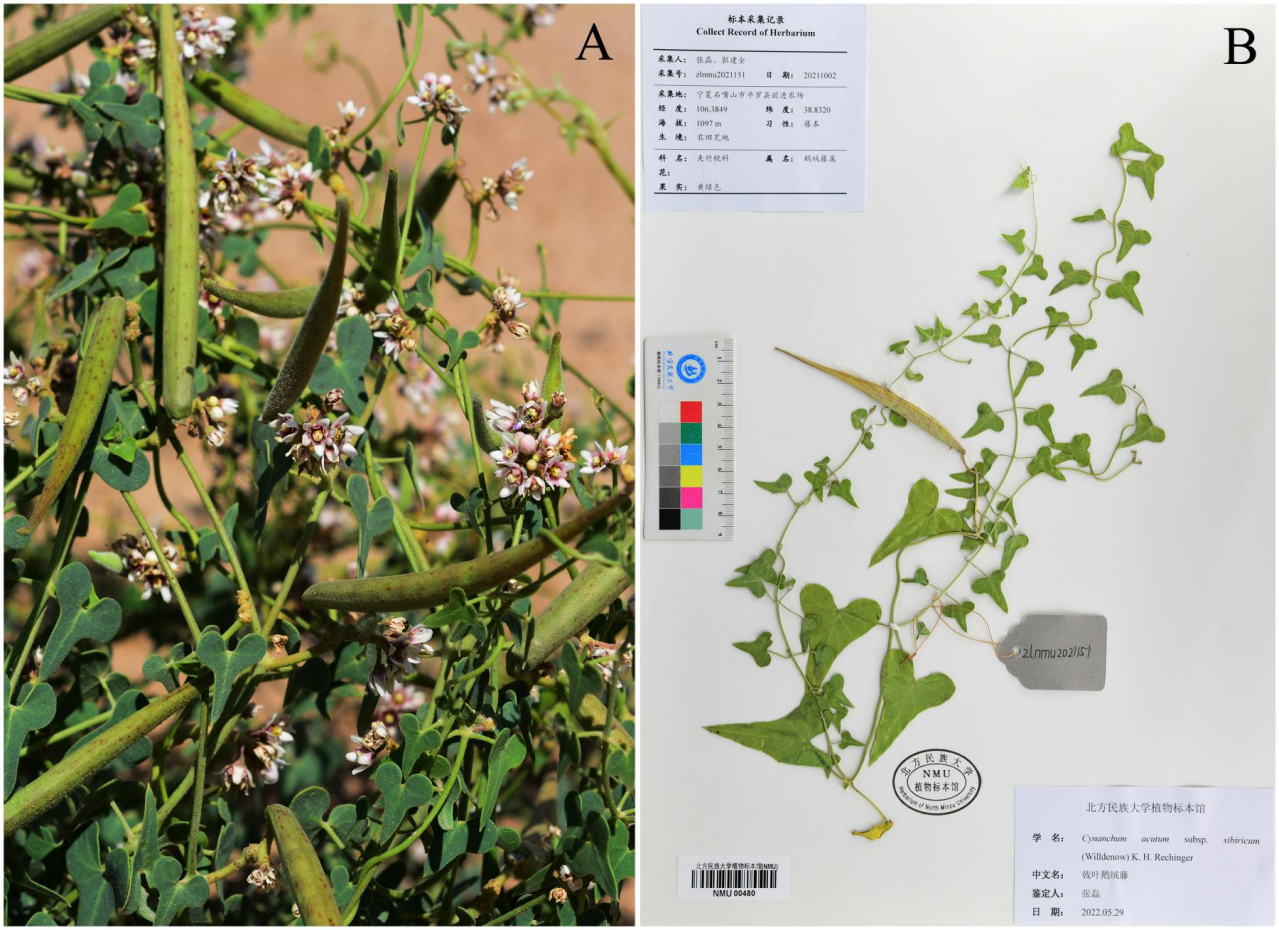
(A) Image of *Cynanchum acutum* subsp. *sibiricum*. (B) Herbarium of *Cynanchum acutum* subsp. *sibiricum* (stems twining, glabrous. leaf blade hastate, 4-6 × 3-4.5 cm, base auriculate, apex long acuminate).

Fresh leaves of *C. acutum* subsp. *sibiricum* were collected from Pingluo (Shizuishan, Ningxia, China; coordinates: 106.3849E, 38.8320 N) (by Lei Zhang: zhangsanshi-0319@163.com) (Figure 1A), and dried with silica gel. The voucher specimen was stored in Herbarium of North Minzu University with an accession number of zlnmu2021151 (Figure 1B).

## 3. Methods

1.0 µg of the leaf sample was measured for DNA preparation. Total genomic DNA was extracted with a modified CTAB method (Doyle and Doyle 1987). NEBNext DNA Library Kit was used to generate the sequencing libraries following the manufacturer’s manual. DNA was fragmented erratically to 350 bp size by shearings. This library was sequenced on the Illumina NovaSeq 6000 platform with 150 bp paired-end read length. We obtained 6.2 Gb high quality pair-end reads for *C. acutum* subsp. *sibiricum*. After removing the adapters, we *de novo* assembled the cp genome of *C. acutum* subsp. *sibiricum* using NOVOPlasty 4.1 (Dierckxsens et al. 2017) using the following parameters: k-mer = 39 and genome range 120,000–200,000 bp. The complete chloroplast genome sequence of *C. auriculatum* (KU900231) was used as a reference. Plann v1.1 (Huang and Cronk 2015) was used to annotate the chloroplast genome and Geneious v11.0.3 (Kearse et al. 2012) was employed to correct the annotation. In order to further clarify the phylogenetic position of *C. acutum* subsp. *sibiricum*, chloroplast genome of nine representative species were obtained from NCBI GenBank to reconstruct the chloroplast genome phylogenetic tree, with *Nerium oleander* being used as an outgroup. All the sequences were aligned using MAFFT v.7.313 (Katoh and Standley 2013) and maximum likelihood phylogenetic analyses were conducted by using RAxML v.8.2.11 (Stamatakis 2014) under GTRCAT model with 1000 bootstrap replicates.

## 4. Results

**Figure 2.**
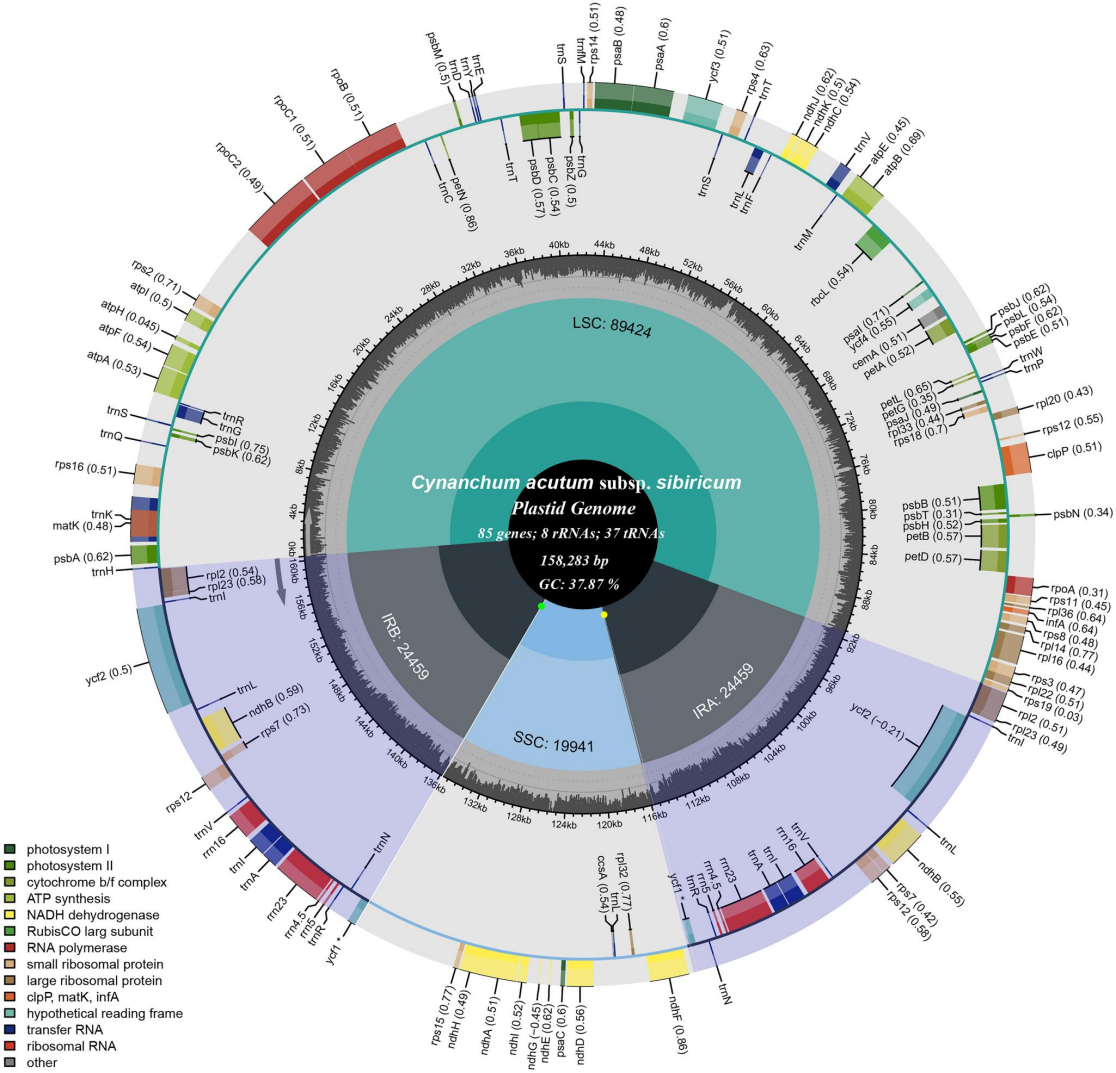
The detailed genome map of *Cynanchum acutum* subsp. *sibiricum* cp genome. The large single copy (LSC), small single copy (SSC) region and two inverted repeat regions (IRA and IRB), and GC content (light gray) are shown in the inside track. Gene models including protein-coding genes, tRNA genes, and rRNA genes are shown with various colored boxes in the outer track.

The total chloroplast genome of *C. acutum* subsp. *sibiricum* (OQ390041) was 158,283 bp in length and depth for average, maximal and minimal were 7893.73 x, 8022 x and 97 x (Supplementary Figure 1), with a typical quadripartite structural organization, consisting of a large single copy (LSC) region of 89,424 bp, two inverted repeat (IR) regions of 24,459 bp and a small single copy (SSC) region of 19,941 bp (Figure 2). The cp genome contained 130 complete genes, including 85 protein-coding genes (85 PCGs), 8 ribosomal RNA genes (8 rRNAs), and 37 tRNA genes (37 tRNAs). Most genes were occurred in a single copy, while 16 genes occurred in double, including four rRNAs (*rrn16*, *rrn23*, *rrn4.5* and *rrn5*), four tRNAs (*trn*A-UGC, *trn*I-CAU*, trn*I-GAU, *trn*L-CAA, *trn*N-GUU, *trn*R-ACG, and *trn*V-GAC), and 5 PCGs (*rpl*23, *rps*7, *ndh*B, *ycf*2 and *rpl*2). Meanwhile, The cp genome contained 13 cis-splicing genes (Supplementary Figure 2) and 1 trans-splicing gene (Supplementary Figure 3). The overall GC content of cp DNA is 37.87%, the respective values for the LSC, SSC, and IR regions were 36.15%, 32.00%, and 43.43%.

**Figure 3.**
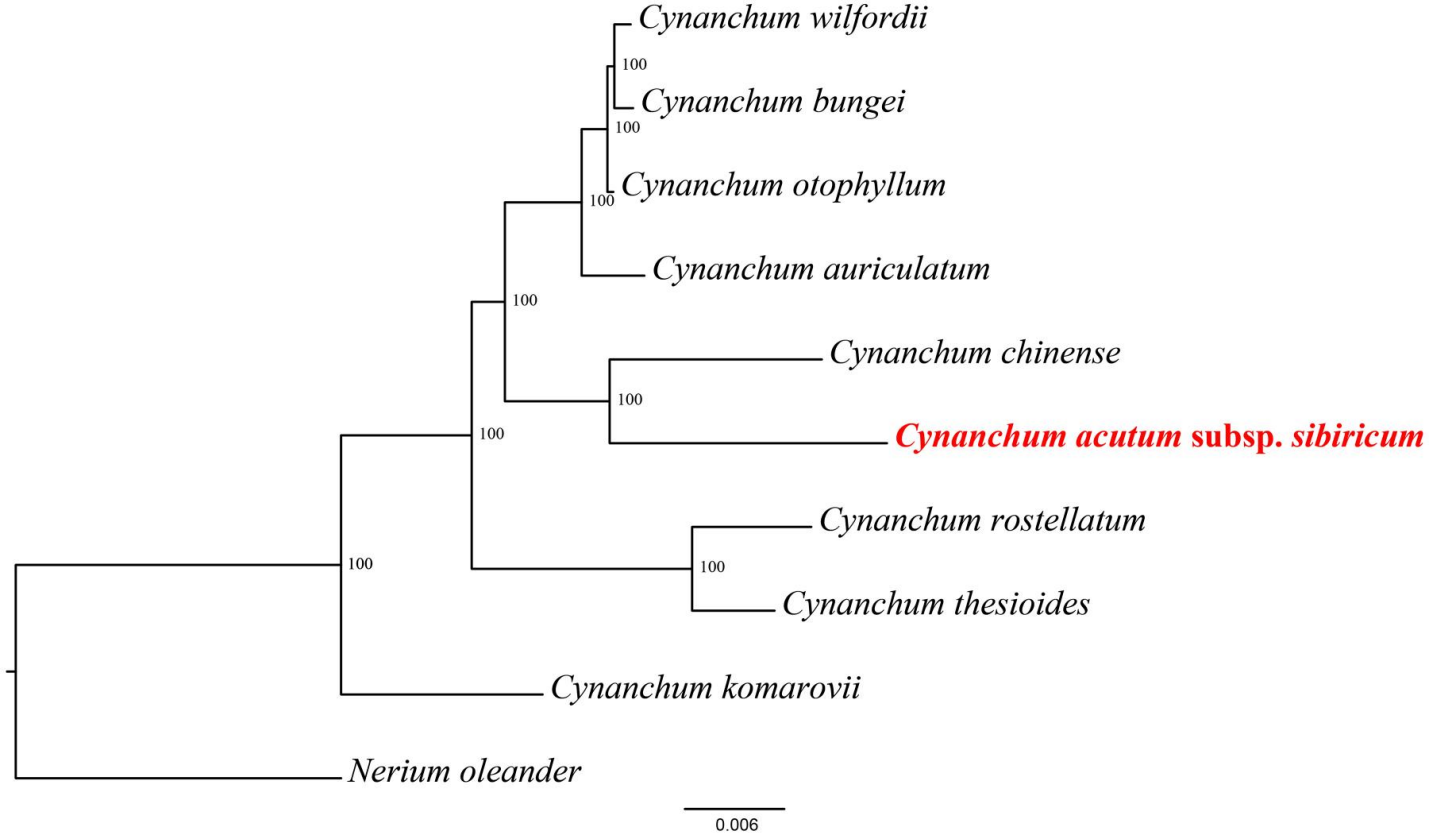
Phylogenetic relationships of *Cynanchum* species using whole chloroplast genome. GenBank accession numbers: *Cynanchum acutum* subsp. *sibiricum* OQ390041, *Cynanchum auriculatum* KU900231 (Yu et al. 2021), *Cynanchum bungei* OK271106 (Pei et al. 2022), *Cynanchum chinense* MW415427 (Chen and Zhang 2022), *Cynanchum otophyllum* OQ587923*, Cynanchum komarovii* ON854661(Liu et al. 2023), *Cynanchum thesioides* MW864598 (Kang et al. 2021), *Cynanchum rostellatum* OQ852689 (Lee et al. 2022), *Cynanchum wilfordii* KX352467 (Hyun-Seung, 2016) and *Nerium oleander* KJ953907.

The phylogenetic tree showed that the species of *Cynanchum* were divided into three subclades (Figure 3). *C. thesioides* and *C. rostellatum* clustered the second subclade, and the *C. chinense* and *C. acutum* subsp. *sibiricum* into another subclade. In addition, *C. acutum* subsp. *sibiricum* has the closest relationship with *C. chinense*.

## 5. Discussion and conclusion

The chloroplast genome of angiosperms provides a powerful tool for species tree estimation, population genetic analysis, chloroplast gene function and chloroplast metabolism analysis, and investigation of species adaptation to extreme environments (Mehmood et al. 2020 a,b,c). Here, we reported the first chloroplast genome for *C. acutum* subsp. *sibiricum.* This cp genome will provide useful genetic resource for further genetic and genomic studies of the genus *Cynanchum* and Apocynaceae.

The phylogenetic tree showed that the species of *Cynanchum* were divided into three subclades. All 8 species and varieties of the ingroup, except *C. komarovii*, comprise a group that supports the monophyly of the *Cynanchum* clade, This phylogenetic result was similar to those of Lee et al. (2022) and Pei et al. (2022). Further studies of the *C. komarovii* is in order, especially to confirm the relation-ships of this species to *Cynanchum*. Increasing the number of cp genomes of *Cynanchum* and Apocynaceae will provide deeper insights into the evolutionary of this ecologically and economically important family.

## Supplementary Material

Supplemental Material

## Data Availability

The sequenced data supporting the findings of this study are openly available in NCBI (https://www.ncbi.nlm.nih.gov/) under the accession no. OQ390041. The associated BioProject, SRA, and Bio-Sample numbers are PRJNA930674, SRR23341524, and SAMN33018279, respectively.
