# Supplementary material for: Characterization of the complete chloroplast genome of Cynanchum acutum subsp. sibiricum (Apocynaceae)
Source: Mitochondrial DNA B Resour. 2023 Sep 19;8(9):993–7. doi: 10.1080/23802359.2023.2256496 (PMC10512800; doi:10.1080/23802359.2023.2256496)
Supplement: Supplemental Material [file TMDN_A_2256496_SM7498.docx]

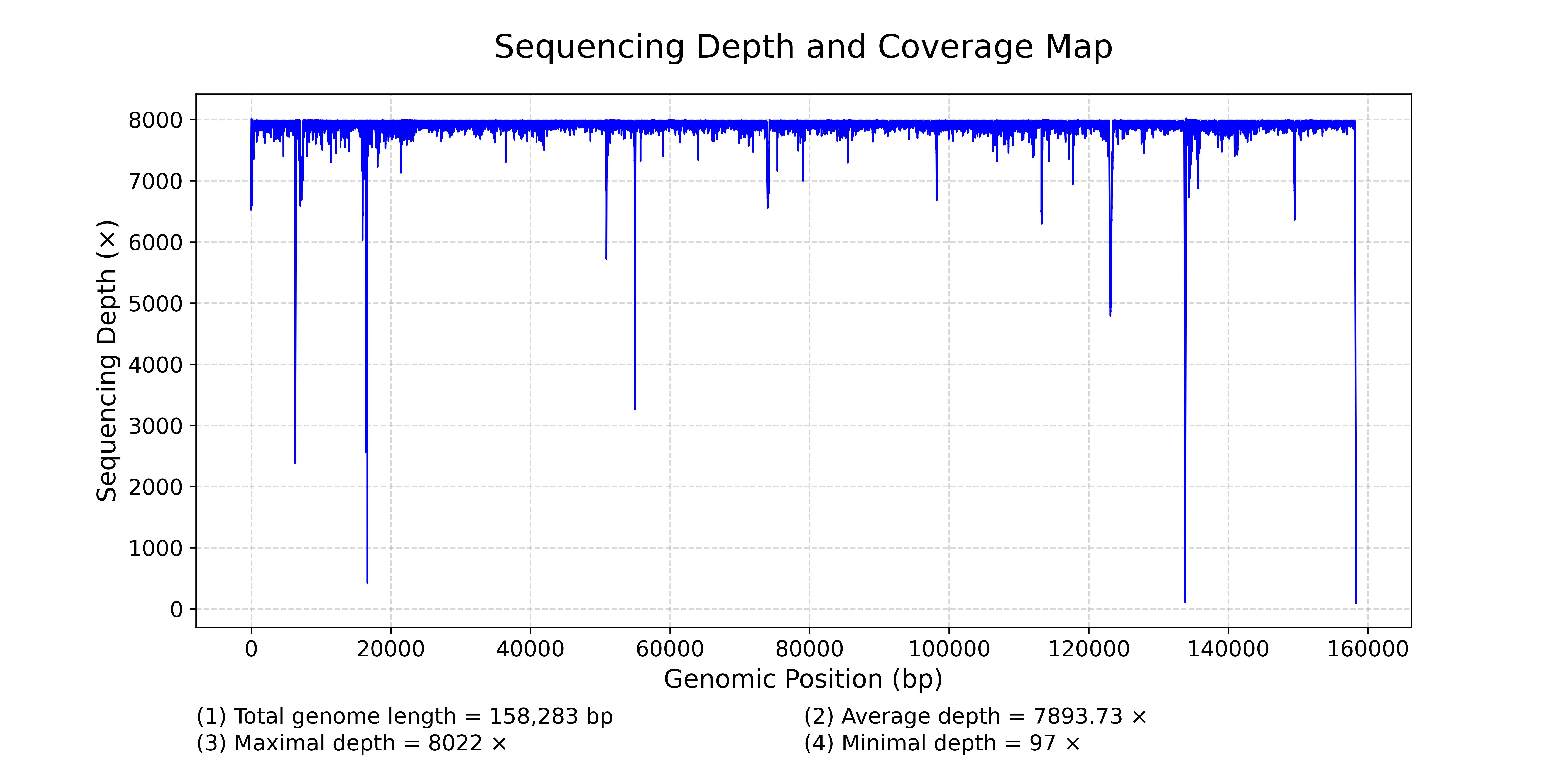


**Supplementary Figure 1** Coverage depth distribution of the *Cynanchum sibiricum* subsp. *sibiricum* cp genome.


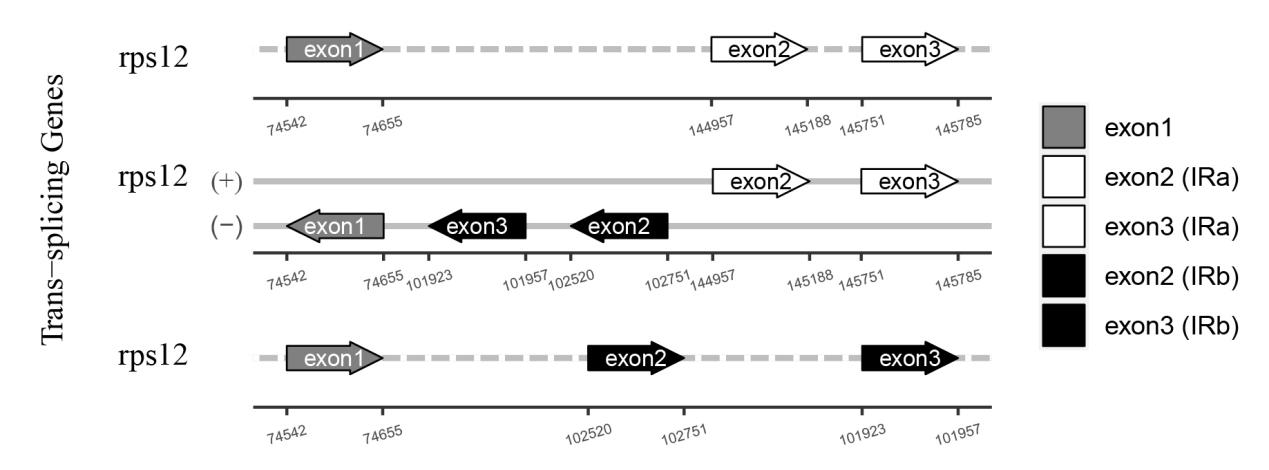


**Supplementary Figure 2**. Structure of trans-splicing genes in the *C. sibiricum* subsp. *sibiricum* cp genome.


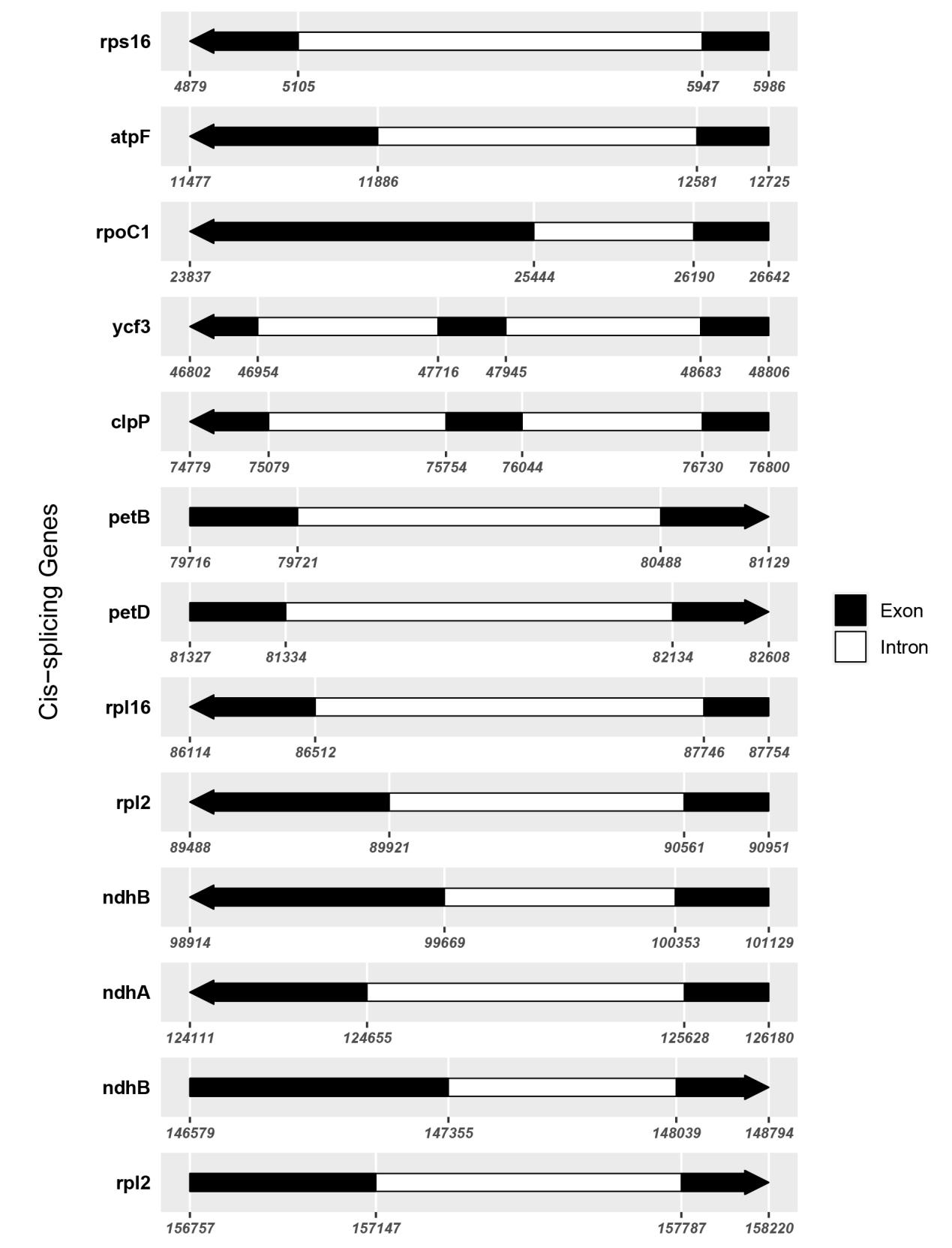


**Supplementary Figure 3** Structure of Cis-splicing genes in the *C. sibiricum* subsp. *sibiricum* cp genome.
